# Diethyl 2,2′-(biphenyl-2,2′-diyldi­oxy)diacetate

**DOI:** 10.1107/S1600536810026863

**Published:** 2010-07-10

**Authors:** Qamar Ali, Itrat Anis, Donald VanDerveer, Muhammad Raza Shah

**Affiliations:** a H.E.J. Research Institute of Chemistry, International Center for Chemical and Biological Sciences, University of Karachi, Karachi 75270, Pakistan; b Department of Chemistry, University of Karachi, Karachi 75270, Pakistan; c Chemistry Department, Clemson University, Clemson, SC 29634-0973, USA

## Abstract

In the title compound, C_20_H_22_O_6_, the mean planes through the benzene rings make a dihedral angle of 59.82 (7)° with each other. Weak inter­molecular C—H⋯O inter­actions together with π–π stacking inter­actions [centroid–centroid distance = 3.830 (1) Å] between benzene rings are observed in the crystal packing.

## Related literature

For related structures, see: Rabnawaz *et al.* (2008 ▶); Ali *et al.* (2008 ▶); Ibad *et al.* (2008 ▶).
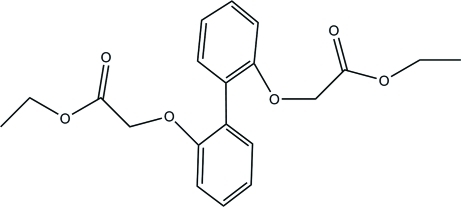


## Experimental

### 

#### Crystal data

C_20_H_22_O_6_
*M* *_r_* = 358.38
Triclinic, 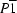

*a* = 7.4683 (13) Å
*b* = 10.8189 (15) Å
*c* = 12.050 (2) Å
α = 104.733 (17)°
β = 95.05 (2)°
γ = 106.897 (14)°
*V* = 887.1 (3) Å^3^
*Z* = 2
Mo *K*α radiation
μ = 0.10 mm^−1^
*T* = 153 K
0.58 × 0.53 × 0.24 mm

#### Data collection

Rigaku AFC-8S diffractometer
Absorption correction: multi-scan (*REQAB*; Jacobson, 1998 ▶) *T*_min_ = 0.945, *T*_max_ = 0.977
6640 measured reflections
3104 independent reflections
2787 reflections with *I* > 2σ(*I*)
*R*_int_ = 0.014

#### Refinement

*R*[*F* ^2^ > 2σ(*F* ^2^)] = 0.039
*wR*(*F* ^2^) = 0.099
*S* = 1.04
3104 reflections
235 parameters
H-atom parameters constrained
Δρ_max_ = 0.18 e Å^−3^
Δρ_min_ = −0.20 e Å^−3^

### 

Data collection: *CrystalClear* (Rigaku/MSC, 2006 ▶); cell refinement: *CrystalClear*; data reduction: *CrystalClear*; program(s) used to solve structure: *SHELXTL* (Sheldrick, 2008 ▶); program(s) used to refine structure: *SHELXTL*; molecular graphics: *SHELXTL*; software used to prepare material for publication: *SHELXTL*.

## Supplementary Material

Crystal structure: contains datablocks I, global. DOI: 10.1107/S1600536810026863/ez2221sup1.cif

Structure factors: contains datablocks I. DOI: 10.1107/S1600536810026863/ez2221Isup2.hkl

Additional supplementary materials:  crystallographic information; 3D view; checkCIF report

## Figures and Tables

**Table 1:** Hydrogen-bond geometry (Å, °)

*D*—H⋯*A*	*D*—H	H⋯*A*	*D*⋯*A*	*D*—H⋯*A*
C4—H4b⋯O1^i^	0.99	2.50	3.472 (2)	168 (1)

Symmetry code: (i) 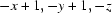.

**Fig. 1.**
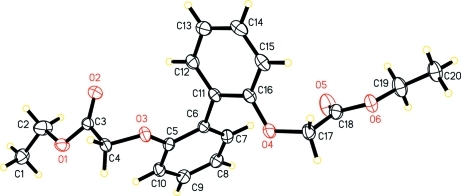
Molecular structure of (I) with atom labels and 30% probability displacement ellipsoids.

## supplementary crystallographic information

### Comment

In the continuation of our work on biphenyl derivatives (Rabnawaz *et al.*, 2008) we report here the synthesis and crystal structure of the title compound, I. The molecular structure and atom-numbering scheme of the title compound are shown in Fig. 1. The molecules are essentially non-planar, with a dihedral angle of 59.82 (7)° between the mean planes through the benzene rings. This is slightly more than the corresponding tert butyl ester analogue of I (Ali *et al.*, 2008), due to the presence of the sterically less crowded ethyl ester group. The key C=O and C—O bond distances are in agreement with those observed in the related hydrazide structure (Ibad *et al.*, 2008). NMR data show that the molecule has a non-crystallographic two-fold rotation axis.

### Experimental

K_2_CO_3_ (414 mg, 3 mmol) and 2,2'-dihydroxybiphenyl (186 mg,1 mmol) in 15 ml of acetone were stirred for 10 minutes, followed by addition of ethyl bromoacetate (314 mg, 3 mmol). The reaction mixture was stirred at room temperature for three hours. The solvent was evaporated under reduced pressure and the residue was dissolved in a mixture of water (50 ml) and dichloromethane (50 ml). The aqueous layer was extracted three times with dichloromethane. The combined organic phases were evaporated under reduced pressure and the solid residue was dissolved in ethanol. Slow evaporation of ethanol gave colorless crystals (736 mg) in 80% yield.

### Refinement

The H atoms were geometrically placed and treated as riding atoms with C—H = 0.96 Å, and *U*_iso_(H) = 1.2 *U*_eq_(parent C-atom) except for methyl H atoms where *U*_iso_(H) = 1.5 *U*_eq_(methyl C-atom).

### Crystal data

**Table:**

C_20_H_22_O_6_	*Z* = 2
*M**_r_* = 358.38	*F*(000) = 380
Triclinic, *P*1	*D*_x_ = 1.342 Mg m^−^^3^
*a* = 7.4683 (13) Å	Mo *K*α radiation, λ = 0.71073 Å
*b* = 10.8189 (15) Å	Cell parameters from 2971 reflections
*c* = 12.050 (2) Å	θ = 3.1–26.3°
α = 104.733 (17)°	µ = 0.10 mm^−^^1^
β = 95.05 (2)°	*T* = 153 K
γ = 106.897 (14)°	Chip, colorless
*V* = 887.1 (3) Å^3^	0.58 × 0.53 × 0.24 mm

### Data collection

**Table:**

Rigaku AFC-8S diffractometer	3104 independent reflections
Radiation source: normal-focus sealed tube	2787 reflections with *I* > 2σ(*I*)
graphite	*R*_int_ = 0.014
Detector resolution: 14.6306 pixels mm^-1^	θ_max_ = 25.1°, θ_min_ = 3.1°
ω scans	*h* = −8→8
Absorption correction: multi-scan (*REQAB*; Jacobson, 1998)	*k* = −12→12
*T*_min_ = 0.945, *T*_max_ = 0.977	*l* = −14→12
6640 measured reflections	

### Refinement

**Table:**

Refinement on *F*^2^	Primary atom site location: structure-invariant direct methods
Least-squares matrix: full	Secondary atom site location: difference Fourier map
*R*[*F*^2^ > 2σ(*F*^2^)] = 0.039	Hydrogen site location: inferred from neighbouring sites
*wR*(*F*^2^) = 0.099	H-atom parameters constrained
*S* = 1.04	*w* = 1/[σ^2^(*F*_o_^2^) + (0.0493*P*)^2^ + 0.3462*P*] where *P* = (*F*_o_^2^ + 2*F*_c_^2^)/3
3104 reflections	(Δ/σ)_max_ < 0.001
235 parameters	Δρ_max_ = 0.18 e Å^−^^3^
0 restraints	Δρ_min_ = −0.20 e Å^−^^3^

### Special details

**Table:**

Geometry. All esds (except the esd in the dihedral angle between two l.s. planes) are estimated using the full covariance matrix. The cell esds are taken into account individually in the estimation of esds in distances, angles and torsion angles; correlations between esds in cell parameters are only used when they are defined by crystal symmetry. An approximate (isotropic) treatment of cell esds is used for estimating esds involving l.s. planes.
Refinement. Refinement of F^2^ against ALL reflections. The weighted R-factor wR and goodness of fit S are based on F^2^, conventional R-factors R are based on F, with F set to zero for negative F^2^. The threshold expression of F^2^ > 2sigma(F^2^) is used only for calculating R-factors(gt) etc. and is not relevant to the choice of reflections for refinement. R-factors based on F^2^ are statistically about twice as large as those based on F, and R- factors based on ALL data will be even larger.

### Fractional atomic coordinates and isotropic or equivalent isotropic displacement parameters (Å^2^)

**Table:**

	*x*	*y*	*z*	*U*_iso_*/*U*_eq_	
O1	0.26152 (13)	0.49153 (10)	−0.09823 (8)	0.0281 (2)	
O2	0.10844 (14)	0.63977 (10)	−0.03410 (9)	0.0289 (3)	
O3	0.37964 (13)	0.73993 (9)	0.17431 (8)	0.0229 (2)	
O4	0.64269 (14)	1.13019 (10)	0.42800 (8)	0.0270 (2)	
O5	0.71807 (19)	1.39716 (12)	0.43450 (10)	0.0455 (3)	
O6	0.74896 (14)	1.45726 (9)	0.62997 (8)	0.0284 (2)	
C1	0.2426 (2)	0.36891 (16)	−0.29568 (13)	0.0315 (3)	
H1A	0.1754	0.3436	−0.3759	0.047*	
H1B	0.2329	0.2872	−0.2723	0.047*	
H1C	0.3768	0.4191	−0.2912	0.047*	
C2	0.1555 (2)	0.45549 (19)	−0.21611 (13)	0.0407 (4)	
H2A	0.1631	0.5380	−0.2395	0.049*	
H2B	0.0201	0.4056	−0.2194	0.049*	
C3	0.22147 (18)	0.58407 (13)	−0.01637 (12)	0.0213 (3)	
C4	0.33426 (18)	0.60535 (13)	0.10190 (11)	0.0218 (3)	
H4A	0.2593	0.5424	0.1406	0.026*	
H4B	0.4532	0.5843	0.0911	0.026*	
C5	0.50568 (17)	0.83979 (13)	0.14036 (11)	0.0197 (3)	
C6	0.51573 (18)	0.97228 (13)	0.19636 (11)	0.0196 (3)	
C7	0.64460 (19)	1.07739 (13)	0.16831 (12)	0.0239 (3)	
H7A	0.6553	1.1682	0.2068	0.029*	
C8	0.75716 (19)	1.05198 (14)	0.08549 (12)	0.0261 (3)	
H8A	0.8428	1.1247	0.0667	0.031*	
C9	0.74388 (19)	0.92018 (15)	0.03053 (12)	0.0267 (3)	
H9A	0.8203	0.9023	−0.0266	0.032*	
C10	0.61985 (19)	0.81358 (14)	0.05811 (12)	0.0244 (3)	
H10A	0.6129	0.7232	0.0210	0.029*	
C11	0.38246 (18)	1.00240 (13)	0.27616 (11)	0.0207 (3)	
C12	0.18639 (19)	0.95132 (13)	0.23529 (12)	0.0242 (3)	
H12A	0.1391	0.8899	0.1593	0.029*	
C13	0.0593 (2)	0.98855 (14)	0.30363 (13)	0.0277 (3)	
H13A	−0.0734	0.9537	0.2745	0.033*	
C14	0.1289 (2)	1.07714 (14)	0.41479 (13)	0.0297 (3)	
H14A	0.0430	1.1041	0.4615	0.036*	
C15	0.3225 (2)	1.12712 (14)	0.45896 (13)	0.0281 (3)	
H15A	0.3685	1.1868	0.5358	0.034*	
C16	0.44882 (19)	1.08923 (13)	0.39009 (12)	0.0223 (3)	
C17	0.7184 (2)	1.23201 (14)	0.53605 (12)	0.0284 (3)	
H17A	0.8479	1.2336	0.5641	0.034*	
H17B	0.6384	1.2107	0.5943	0.034*	
C18	0.72672 (19)	1.36982 (14)	0.52501 (12)	0.0263 (3)	
C19	0.7734 (2)	1.59703 (14)	0.63189 (14)	0.0349 (4)	
H19A	0.6748	1.5994	0.5726	0.042*	
H19B	0.8998	1.6391	0.6141	0.042*	
C20	0.7560 (2)	1.67243 (15)	0.75077 (14)	0.0352 (4)	
H20A	0.7719	1.7665	0.7541	0.053*	
H20B	0.8544	1.6699	0.8088	0.053*	
H20C	0.6303	1.6303	0.7674	0.053*	

### Atomic displacement parameters (Å^2^)

**Table:**

	*U*^11^	*U*^22^	*U*^33^	*U*^12^	*U*^13^	*U*^23^
O1	0.0283 (5)	0.0326 (6)	0.0214 (5)	0.0160 (4)	0.0003 (4)	−0.0010 (4)
O2	0.0284 (5)	0.0264 (5)	0.0338 (6)	0.0133 (4)	0.0033 (4)	0.0075 (4)
O3	0.0279 (5)	0.0169 (5)	0.0224 (5)	0.0059 (4)	0.0092 (4)	0.0032 (4)
O4	0.0303 (5)	0.0263 (5)	0.0210 (5)	0.0131 (4)	0.0000 (4)	−0.0019 (4)
O5	0.0766 (9)	0.0358 (6)	0.0258 (6)	0.0188 (6)	0.0108 (6)	0.0107 (5)
O6	0.0422 (6)	0.0211 (5)	0.0213 (5)	0.0123 (4)	0.0035 (4)	0.0036 (4)
C1	0.0309 (7)	0.0337 (8)	0.0238 (8)	0.0074 (6)	0.0037 (6)	0.0020 (6)
C2	0.0376 (8)	0.0587 (11)	0.0225 (8)	0.0246 (8)	−0.0030 (6)	−0.0013 (7)
C3	0.0195 (6)	0.0172 (6)	0.0270 (7)	0.0054 (5)	0.0068 (5)	0.0058 (5)
C4	0.0249 (7)	0.0167 (6)	0.0235 (7)	0.0077 (5)	0.0054 (5)	0.0039 (5)
C5	0.0204 (6)	0.0194 (6)	0.0174 (6)	0.0047 (5)	0.0021 (5)	0.0046 (5)
C6	0.0210 (6)	0.0202 (6)	0.0170 (6)	0.0075 (5)	0.0013 (5)	0.0041 (5)
C7	0.0269 (7)	0.0203 (7)	0.0225 (7)	0.0067 (5)	0.0021 (5)	0.0047 (5)
C8	0.0241 (7)	0.0269 (7)	0.0254 (7)	0.0039 (5)	0.0052 (5)	0.0093 (6)
C9	0.0247 (7)	0.0315 (7)	0.0212 (7)	0.0068 (6)	0.0082 (5)	0.0044 (6)
C10	0.0257 (7)	0.0221 (7)	0.0219 (7)	0.0071 (5)	0.0053 (5)	0.0008 (5)
C11	0.0265 (7)	0.0172 (6)	0.0212 (7)	0.0094 (5)	0.0063 (5)	0.0072 (5)
C12	0.0288 (7)	0.0193 (6)	0.0265 (7)	0.0088 (5)	0.0067 (6)	0.0085 (5)
C13	0.0268 (7)	0.0240 (7)	0.0373 (8)	0.0098 (6)	0.0119 (6)	0.0138 (6)
C14	0.0370 (8)	0.0264 (7)	0.0356 (8)	0.0171 (6)	0.0198 (6)	0.0138 (6)
C15	0.0399 (8)	0.0242 (7)	0.0241 (7)	0.0153 (6)	0.0117 (6)	0.0063 (6)
C16	0.0290 (7)	0.0189 (6)	0.0217 (7)	0.0110 (5)	0.0054 (5)	0.0067 (5)
C17	0.0362 (8)	0.0254 (7)	0.0206 (7)	0.0137 (6)	−0.0020 (6)	0.0000 (6)
C18	0.0289 (7)	0.0281 (7)	0.0200 (7)	0.0096 (6)	0.0026 (5)	0.0040 (6)
C19	0.0524 (9)	0.0208 (7)	0.0330 (9)	0.0128 (7)	0.0078 (7)	0.0094 (6)
C20	0.0516 (9)	0.0201 (7)	0.0318 (8)	0.0105 (7)	0.0062 (7)	0.0056 (6)

### Geometric parameters (Å, °)

**Table:**

O1—C3	1.3374 (16)	C8—C9	1.381 (2)
O1—C2	1.4618 (18)	C8—H8A	0.9500
O2—C3	1.2044 (16)	C9—C10	1.388 (2)
O3—C5	1.3864 (16)	C9—H9A	0.9500
O3—C4	1.4181 (16)	C10—H10A	0.9500
O4—C16	1.3816 (17)	C11—C12	1.399 (2)
O4—C17	1.4167 (17)	C11—C16	1.402 (2)
O5—C18	1.2017 (18)	C12—C13	1.3900 (19)
O6—C18	1.3362 (17)	C12—H12A	0.9500
O6—C19	1.4634 (17)	C13—C14	1.385 (2)
C1—C2	1.492 (2)	C13—H13A	0.9500
C1—H1A	0.9800	C14—C15	1.389 (2)
C1—H1B	0.9800	C14—H14A	0.9500
C1—H1C	0.9800	C15—C16	1.3913 (19)
C2—H2A	0.9900	C15—H15A	0.9500
C2—H2B	0.9900	C17—C18	1.514 (2)
C3—C4	1.5175 (19)	C17—H17A	0.9900
C4—H4A	0.9900	C17—H17B	0.9900
C4—H4B	0.9900	C19—C20	1.495 (2)
C5—C10	1.3916 (18)	C19—H19A	0.9900
C5—C6	1.3971 (18)	C19—H19B	0.9900
C6—C7	1.3966 (19)	C20—H20A	0.9800
C6—C11	1.4897 (18)	C20—H20B	0.9800
C7—C8	1.386 (2)	C20—H20C	0.9800
C7—H7A	0.9500		
			
C3—O1—C2	116.34 (11)	C9—C10—H10A	120.2
C5—O3—C4	116.66 (10)	C5—C10—H10A	120.2
C16—O4—C17	117.43 (11)	C12—C11—C16	118.38 (12)
C18—O6—C19	115.91 (11)	C12—C11—C6	119.93 (12)
C2—C1—H1A	109.5	C16—C11—C6	121.54 (12)
C2—C1—H1B	109.5	C13—C12—C11	121.36 (13)
H1A—C1—H1B	109.5	C13—C12—H12A	119.3
C2—C1—H1C	109.5	C11—C12—H12A	119.3
H1A—C1—H1C	109.5	C14—C13—C12	119.08 (13)
H1B—C1—H1C	109.5	C14—C13—H13A	120.5
O1—C2—C1	107.38 (12)	C12—C13—H13A	120.5
O1—C2—H2A	110.2	C13—C14—C15	120.95 (13)
C1—C2—H2A	110.2	C13—C14—H14A	119.5
O1—C2—H2B	110.2	C15—C14—H14A	119.5
C1—C2—H2B	110.2	C14—C15—C16	119.63 (14)
H2A—C2—H2B	108.5	C14—C15—H15A	120.2
O2—C3—O1	124.57 (13)	C16—C15—H15A	120.2
O2—C3—C4	125.14 (12)	O4—C16—C15	123.64 (12)
O1—C3—C4	110.27 (11)	O4—C16—C11	115.79 (12)
O3—C4—C3	111.80 (11)	C15—C16—C11	120.56 (13)
O3—C4—H4A	109.3	O4—C17—C18	111.40 (12)
C3—C4—H4A	109.3	O4—C17—H17A	109.3
O3—C4—H4B	109.3	C18—C17—H17A	109.3
C3—C4—H4B	109.3	O4—C17—H17B	109.3
H4A—C4—H4B	107.9	C18—C17—H17B	109.3
O3—C5—C10	123.59 (12)	H17A—C17—H17B	108.0
O3—C5—C6	115.63 (11)	O5—C18—O6	124.81 (13)
C10—C5—C6	120.77 (12)	O5—C18—C17	124.61 (13)
C7—C6—C5	118.18 (12)	O6—C18—C17	110.57 (12)
C7—C6—C11	119.86 (12)	O6—C19—C20	108.07 (12)
C5—C6—C11	121.75 (12)	O6—C19—H19A	110.1
C8—C7—C6	121.36 (13)	C20—C19—H19A	110.1
C8—C7—H7A	119.3	O6—C19—H19B	110.1
C6—C7—H7A	119.3	C20—C19—H19B	110.1
C9—C8—C7	119.49 (13)	H19A—C19—H19B	108.4
C9—C8—H8A	120.3	C19—C20—H20A	109.5
C7—C8—H8A	120.3	C19—C20—H20B	109.5
C8—C9—C10	120.55 (13)	H20A—C20—H20B	109.5
C8—C9—H9A	119.7	C19—C20—H20C	109.5
C10—C9—H9A	119.7	H20A—C20—H20C	109.5
C9—C10—C5	119.64 (13)	H20B—C20—H20C	109.5
			
C3—O1—C2—C1	171.24 (12)	C7—C6—C11—C16	−58.95 (18)
C2—O1—C3—O2	−1.3 (2)	C5—C6—C11—C16	126.46 (14)
C2—O1—C3—C4	176.98 (12)	C16—C11—C12—C13	2.17 (19)
C5—O3—C4—C3	−67.26 (14)	C6—C11—C12—C13	−173.39 (12)
O2—C3—C4—O3	−33.27 (18)	C11—C12—C13—C14	−0.6 (2)
O1—C3—C4—O3	148.41 (11)	C12—C13—C14—C15	−1.0 (2)
C4—O3—C5—C10	−15.26 (18)	C13—C14—C15—C16	0.9 (2)
C4—O3—C5—C6	166.12 (11)	C17—O4—C16—C15	−9.89 (19)
O3—C5—C6—C7	178.06 (11)	C17—O4—C16—C11	171.55 (11)
C10—C5—C6—C7	−0.60 (19)	C14—C15—C16—O4	−177.75 (12)
O3—C5—C6—C11	−7.26 (18)	C14—C15—C16—C11	0.8 (2)
C10—C5—C6—C11	174.08 (12)	C12—C11—C16—O4	176.37 (11)
C5—C6—C7—C8	1.4 (2)	C6—C11—C16—O4	−8.15 (18)
C11—C6—C7—C8	−173.42 (12)	C12—C11—C16—C15	−2.24 (19)
C6—C7—C8—C9	−0.9 (2)	C6—C11—C16—C15	173.24 (12)
C7—C8—C9—C10	−0.4 (2)	C16—O4—C17—C18	−75.23 (15)
C8—C9—C10—C5	1.1 (2)	C19—O6—C18—O5	−3.1 (2)
O3—C5—C10—C9	−179.17 (12)	C19—O6—C18—C17	175.61 (12)
C6—C5—C10—C9	−0.6 (2)	O4—C17—C18—O5	−17.8 (2)
C7—C6—C11—C12	116.47 (14)	O4—C17—C18—O6	163.46 (11)
C5—C6—C11—C12	−58.13 (18)	C18—O6—C19—C20	168.59 (13)

### Hydrogen-bond geometry (Å, °)

**Table:**

*D*—H···*A*	*D*—H	H···*A*	*D*···*A*	*D*—H···*A*
C4—H4b···O1^i^	0.99	2.50	3.472 (2)	167.7 (1)

Symmetry codes: (i) −*x*+1, −*y*+1, −*z*.
